# Supplementary material for: Elastocapillary cleaning of twisted bilayer graphene interfaces
Source: Nat Commun. 2021 Aug 20;12:5069. doi: 10.1038/s41467-021-25302-2 (PMC8379234; doi:10.1038/s41467-021-25302-2)
Supplement: Supplementary file 1 — Supplementary Information [file 41467_2021_25302_MOESM1_ESM.docx]

**Elastocapillary cleaning of twisted bilayer graphene interfaces**

Yuan Hou^1,2, §^, Zhaohe Dai^3,§^, Shuai Zhang^4,5,§^, Shizhe Feng^4^, Guorui Wang^1,2^, Luqi Liu^1,*^, Zhiping Xu^4*^, Qunyang Li^4,5,*^, Zhong Zhang^1,2,*^

^1^CAS Key Laboratory of Nanosystem and Hierarchical Fabrication, National Center for Nanoscience and Technology, Beijing, 100190, P.R. China

^2^CAS Key Laboratory of Mechanical Behavior and Design of Materials, Department of Modern Mechanics, University of Science and Technology of China, Hefei, 230026, P. R. China

^3^Department of Aerospace Engineering and Engineering Mechanics, The University of Texas at Austin, Austin, TX 78712, USA

^4^Applied Mechanics Laboratory, Department of Engineering Mechanics, and Center for Nano and Micro Mechanics, Tsinghua University, Beijing, 100084, P. R. China

^5^State Key Laboratory of Tribology, Tsinghua University, Beijing, 100084, P. R. China

*Correspondence and requests for materials should be addressed to L.L. (Email: [liulq@nanoctr.cn](mailto:liulq@nanoctr.cn)), or Z. X (Email: [xuzp@tsinghua.edu.cn](mailto:xuzp@tsinghua.edu.cn)) or Q.L. (Email: [qunyang@tsinghua.edu.cn](mailto:qunyang@tsinghua.edu.cn)) or Z.Z. (Email: [zhong.zhang@nanoctr.cn](mailto:zhong.zhang@nanoctr.cn)).

^§^These authors contribute equally.

**This supplementary material contains the following sections:**

### **1. Experimental Details (Supplementary Figure 1-6 on Page 3-8)**

This section provides experimental details about how the water-filled nanopockets are prepared along with the twisted bilayer graphene, and how their shape is characterized by lateral force AFM. Such information would be helpful for follow-up works.

### **2. Twisting and Stretching (Supplementary Figures 7-10 on Page 9-12)**

This section provides details about how the twisting angle of a tBLG is determined with the aid of AFM and Raman spectroscopy. Since the stretch to these nanopockets is introduced by pressurizing their host bilayer, typical profiles of the pressurized bilayer are also provided, together with the shape of nanopockets when their host bilayer subjects to various pressurized heights.

### **3. Ethanol nanopockets and self-coalescence (Supplementary Figures 11-15 on Page 13-17)**

This section provides the experimental results of the ethanol nanopockets and the details of the coalescence process.

### **4. Molecular dynamics simulations (Supplementary Figures 16-17 on Page 18-19)**

This section provides additional molecular dynamics simulation snapshots.

### **5. A Simple Analysis (Supplementary Figures 18-19 on Page 20-22)**

This section focuses on a simplified analysis that allows analytical expressions for the geometric features of nanopockets before wrinkling instabilities initiate.

### **6. Detailed Calculations (Supplementary Figure 20-21 on Page 23-26)**

This section calibrates the analytic results in the simple analysis via a more rigorous numerically-based analysis as well as provides numerical results on the configuration after instabilities initiate.

### **1. Experimental Details**

**Supplementary Figure 1.** **Preparation of suspended tBLG samples.** (a-f) Schematics of the fabrication processes of suspended tBLG with water nanopockets being trapped at the bilayer interface. (g) A typical optical image of a tBLG on pre-patterned SiO_2_/Si substrate. (h) AFM height image of a tBLG suspending over SiO_2_ pre-patterned with holes. (i) AFM lateral force image of a nanopocket. Scale bars: 10 μm in (g), 1 μm in (h), and 20 nm in (i).

**Supplementary Figure 2. The technique for the extraction of the shape of nanopockets.** The height image (a) and lateral force image (b) of a nanopocket in the trace scan. A uniform moiré pattern can be seen outside the nanopocket. (c) The height signal in the trace scan (blue) and retrace scan (red) measured along the solid white line in (a). The lines represent the scan directions. (d) The lateral force signal in a trace scan (blue) and retrace scan (red) measured along the white line in (b). We identify the edge of a nanopocket at the location where the lateral force signal starts losing/gaining periodicity as labeled by the solid black lines in (d). Scale bars: 30 nm in (a) and (b).

**Supplementary Figure 3**. **Extracted profile details of nanopockets.** Typical 3D morphology of nanopockets before (a) and after coalescence (b). Line-scan height curves of nanopockets before (c) and after coalescence (d).

**Supplementary Figure 4. Compressibility of nanopockets.** Histogram of the volume change of nanopockets that is defined by the ratio of the total volume of the nanopockets after and before the coalescence. The volume of a nanopocket is calculated by reconstructing its 3D morphology via Matlab. The solid blue line is the Gaussian distribution curve of the experimental data based on 18 groups of measurements.

**Supplementary Figure 5. Evidence for the trapped water molecules.** Fourier transform infrared (FTIR) spectrum of tBLG entrapped with heavy water (D_2_O) recorded by Spotlight 200i, Perkin Elmer. The detailed experimental process is the same as those in Fig. S1 while H_2_O is replaced by D_2_O. The peaks at 2340 cm^-1^ and 2360 cm^-1^ correspond to the stretch mode of the O-D bond^1^. The inset shows the optical image of the sample on the SiO_2_/Si substrate (coated with 60-nm-thick Au). The dashed square shows the acquisition region of the FTIR spectrum. Scale bar: 5 μm.

**Supplementary Figure 6. Shape of nanopockets with various radii.** We show typical 3D morphology of round nanopockets trapped within tBLG with various twist angles, on which our analysis in Fig. 2 is based.

### **2. Twisting and Stretching**

The twisting angle $\theta$ is determined by AFM scans if $\theta\lesssim8^{\circ}$ while estimated by Raman spectroscopy otherwise.

**Supplementary Figure 7**. **Determination of moiré periodicity of tBLG**. (a) Sketch of moiré patterns in which $\theta$ is the twisting angle and $L$ is the length for the periodic pattern^2^. (b) Lateral force AFM images of moiré pattern. The twisting angles of samples are ~0.6°, ~1.5°, ~7.3°, which are determined according to the periodicity shown in (c). Scale bars: (b) 20 nm.

**Supplementary Figure 8. Raman spectra of tBLG with different twist angles**. We employed a Renishaw system with an incident wavelength of 514 nm; The spatial resolution was ~1 μm; The laser intensity was kept below 0.5 mW to avoid local heating induced by the laser. We use Raman spectroscopy to identify the twisting angle $\theta$ for the samples whose moiré patterns are too small to be precisely detected by AFM (i.e.$\theta\gtrsim8^{\circ}$). (i) When $8^{\circ}<\theta<9^{\circ}$: The R’ band can be observed. (ii) When $10^{\circ}<\theta<15^{\circ}$: the intensity of the G band is ∼10-fold of that of the 2D band. Such enhancement implies the strong interlayer interaction of *π*-bond electrons and the emergence of VHSs^3^. The R band can be detected, and its frequency decreases linearly with $\theta$,^4^ following which we identified our 10°-13° twisted samples according to the wavenumbers of R band ($>1450 \mathrm{cm}^{-1}$). Note that the Raman G band that locates at ∼1580 cm^−1^, originates from a conventional first-order Raman scattering process and corresponds to the in-plane, zone center, doubly degenerate phonon mode (transverse (TO) and longitudinal (LO) optical) with E_2g_ symmetry^5^. While the 2D band is associated with a second-order process, involving two iTO phonons near the K point for the 2D band^6^.

**Supplementary Figure 9.** Typical height profiles of pressurized tBLG drumheads. The radius is fixed to be ~1.5 μm.

**Supplementary Figure 10. AFM images showing the coalescence of nanopockets.** From left to middle, the host bilayer drumhead is almost flat, and the coalescence is actuated by AFM tip stimuli. After merging, the wrinkles around pockets still exist. From middle to the right, the host drumhead is pressurized with an aspect ratio of ~0.045. This process also motivated the coalescence of nanopockets so that the drumhead surface appears cleaner. In this case, the pretension is introduced by the pressure, and the wrinkles get suppressed. Scale bars: 200 nm.

### **3. Ethanol nanopockets and self-coalescence**

**Supplementary Figure 11. Comparison between water and ethanol nanopockets.** AFM images showing the wrinkles around water (a) and ethanol nanopockets (b). (c) Aspect ratios of water and ethanol nanopockets. Scale bars: 100 nm. We have performed experiments on ethanol contaminants. Note that the twist angle of nanopockets is $\sim0.6^{\circ}$. We found that the aspect ratios of ethanol nanopockets are 0.106 ± 0.011, which is 0.108 ± 0.032 for water nanopockets. The reason for this may be that the graphene-graphene interfacial energy may dominate over the graphene-water or graphene-ethanol interfacial energy. For each nanopocket, the radius a and bubble height h were taken along three directions, from which the average values of *a* and *h/a* as well as x(y)-error bars were determined.

**Supplementary Figure 12. Aspect ratios of ethanol nanopockets in response to stretching.** (a) The dependence of the aspect ratio of nanopockets on the aspect ratio of their host drumheads. Nanopockets whose aspect ratios are in the shaded region are predicted to subject to hoop compression and hence elastic instabilities near the edge. Solid markers denote nanopockets with wrinkles while the crossed markers are samples in which the wrinkling is suppressed by the pretension. The aspect ratio is measured based on nanopockets located in the center of tBLG drumhead so that the stretch to the nanopockets caused by the pressurization is relatively uniform. For each nanopocket, the radius a and bubble height h were taken along three directions, from which the average value of *h/a* as well as y-error bars were determined. (b-d) AFM lateral force images of nanopockets without and with pretension due to the pressurization of their host drumhead. Scale bars: 50 nm.

**Supplementary Figure 13.** **Coalescence of nanopockets.** AFM images of nanopockets on tBLG without pretension. The white arrows correspond to the direction of the mechanical stimuli. The black arrows indicate the moving direction of the nanopocket-B. (a) The initial configurations of four nanopockets. (b) The configurations after the first mechanical stimuli on the nanopocket-B. (c) The configuration after the second mechanical stimuli on the nanopocket-B. (d) The configuration after the mechanical stimuli on the nanopocket-C. Scale bars: 50 nm.

**Supplementary Figure 14.** **Characterization of the wrinkled configurations.** (a-c) Lateral force images of graphene nanopockets show that the wrinkles do not behave as channels before the nanopockets merge spontaneously. (d-e) The cross-sectional height profiles of wrinkled regions. Scale bar: (a) 200 nm, (b)(c) 50 nm.

**Supplementary Figure 15.** **Characterization of roundness of nanopockets.** (a-b) The lateral force images and schematics of adjacent and single nanopockets. (c-d) Roundness of adjacent/single nanopockets with and without pretension. (e) The relationship between the roundness of nanopockets and the distance between nanopockets. Roundness is defined as the ratio of inscribed circle (*r*) and circumscribed circle (*R*) of the nanopocket. From the lateral force images of nanopockets, we found that the wrinkles around the two nanopockets are overlapped to form a connecting structure (we defined as “adjacent nanopockets”). The nanopockets far away from each other are defined as the “single nanopockets”. The normalized roundness of the adjacent and single nanopockets with different sizes and pretensions are calculated. Here, the normalized roundness is expressed as *r*/*R*. The results indicate that i) the normalized roundness of nanopockets is insensitive to the radii of nanopockets; ii) the normalized roundness of adjacent nanopockets is smaller than that of single nanopockets; iii) the pretension can increase the roundness of nanopockets. Since the roundness represents the proximity of nanopocket’s shape to the standard circle, we infer that both the distance between nanopockets and the pretension can influence the shape of nanopockets. To correlate the non-roundness of neighboring nanopockets to the distance, we define the normalized distance as *d/*(*R__L_+R__S_*). To clarify the result, we focus on the smaller nanopockets (*R__S_*, *r__S_*). It does suggest clearly that as the distance between nanopockets decreases, the roundness of the smaller nanopockets shows a decrease trend. Due to the symmetry breaking of the nanopocket is caused by the elastocapillary interaction, the result indicates that the range of interaction is limited by the distance between nanopockets. Scale bar: (a) (b) 30 nm.

### **4. Molecular dynamics simulations**

**
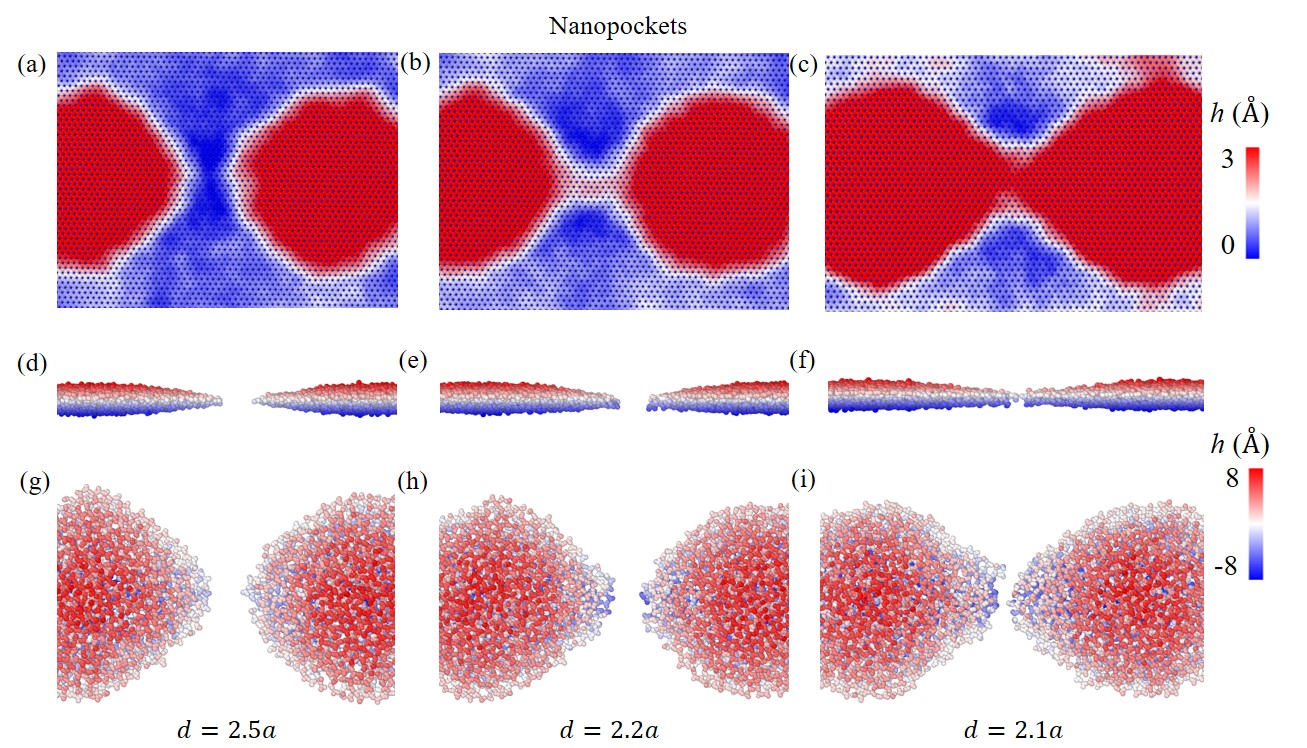
**

**Supplementary Figure 16.** Molecular simulation snapshots showing the coalescence process of nanopockets intercalated in a graphene bilayer without pretension. (a-c) The height maps of two approaching nanopockets. (d-f) The side views of nanodroplets. (g-i) The top views of nanodroplets. The MD simulations show that when the nanodroplets approaching to each other, the morphologies of nanodroplets become asymmetric. The edges of nanodroplets become flatter because the long-range interaction increases, which is consistent with our theoretical analysis and experimental results.


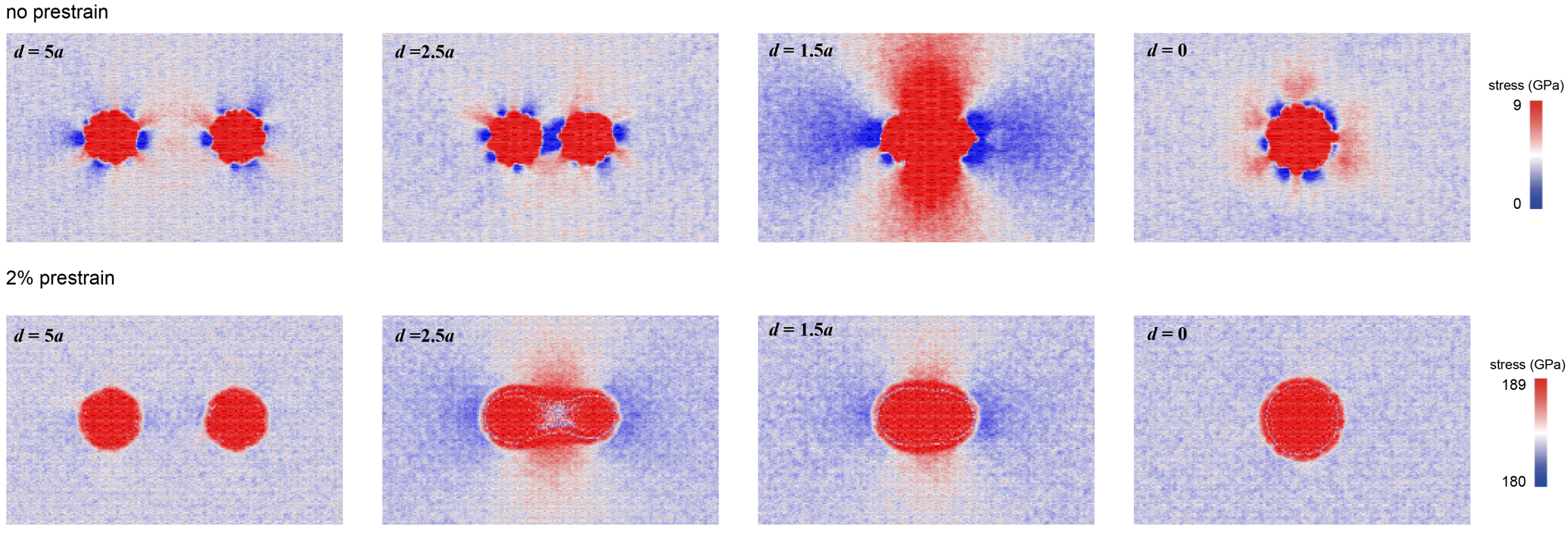


**Supplementary Figure 17.** Stress distributions during coalescence of nanopockets with and without pretension. The averaged stress ($\sigma_{1}+\sigma_{2}+\sigma_{3})/3$ is calculated (the subscripts 1,2,3 denote the stress component along in-plane (1,2) and out-of-plane (3) directions, respectively), showing that the wrinkled features identified in the absence of pretension are reduced by applying pretension of 2%.

### **5. A Simple Analysis**

**Supplementary Figure 18.** Schematic of a nanopocket with its geometric notations labeled, including height $h$*,* radius $a$, contact angle $\beta_{0}$*.*

To make analytical progress, we first consider a situation without wrinkles. Similar to previous studies^7, 8^, we assume that the top profile of the nanopocket could be characterized by

$w\left( r \right)=h\left( 1-\frac{r^{2}}{a^{2}} \right)$. (S5.1)

The bottom profile can be obtained simply by the reflection of $w\to-w$ since the gravity effect is negligible, and the nanopocket is both circularly and horizontally symmetrical. The volume of the nanopocket can then be calculated,

$V=\pi a^{2}h$. (S5.2)

The profile in (S5.1) allows the direct solution to the in-plane equilibrium of the membrane limit of Föppl–von Kármán (FvK) equations in terms of displacements^4^,

$u(r)=\frac{3-\nu}{4}\frac{h^{2}}{a}\left( \frac{r}{a}-\left( \frac{r}{a} \right)^{3} \right)+u_{s}\frac{r}{a}$, (S5.3)

where $u$ is the in-plane displacement and $\nu$ is the Poisson’s ratio, and $u_{s}$ is a constant that is related to the radial displacement at the edge of the nanopocket. The radial and circumferential strain fields can be derived based on the kinematics, i.e.,

$\epsilon_{r}=\frac{du}{dr}{+\frac{1}{2}\left( \frac{dw}{dr} \right)}^{2}$ and $\epsilon_{\theta}=\frac{u}{r}$. (S5.4)

To determine $u_{s}$, we move on to the region outside of the nanopocket ($r>a$), where the two graphene sheets are clamped by their vdW interactions. It is natural to assume a vanished friction between two relatively twisted sheets so that the plane-stress Lame solution^9^ can be adopted:

$N_{r}=\frac{A}{r^{2}}+T_{\mathrm{pre}}$ and $N_{\theta}=-\frac{A}{r^{2}}+T_{\mathrm{pre}}$*,* (S5.5)

where $N_{r}=\sigma_{r}t$ and $N_{\theta}{=\sigma}_{\theta}t$; $\sigma_{r}$ and $\sigma_{\theta}$ are, respectively, radial and circumferential stresses; $t$ is the membrane thickness; $A$ is a constant to be determined; $T_{\mathrm{pre}}$ is the pre-tension applied to the nanopocket through the drumhead device. The radial stress and displacement have to be continuous across the nanopocket edge; Through (S5.3-5.5) and linear Hooke’s law, we obtain

$A=\frac{E_{2D}h^{2}}{4}$ and $u_{s}=\frac{\left( 1-\nu\right)aT_{\mathrm{pre}}}{E_{2D}}-\frac{\left( 1+\nu\right)h^{2}}{4a}$*.* (S5.6)

We enforce the force balance at the contact line to ensure the equilibrium of the system,^[[1]](#footnote-1)^

$\cos\beta_{0}=\frac{N_{r}^{+}+\gamma_{\mathrm{gg}}/2}{N_{r}^{-}+\gamma_{\mathrm{gl}}}$. (S5.7)

where $\beta_{0}$ is the contact angle as defined in Fig. S18, $\gamma_{\mathrm{gg}}$ and $\gamma_{\mathrm{gl}}$ are the energy density (per unit area) for the twisted graphene-graphene interface and the graphene-liquid interface, respectively. In experiments, it is more accurate to convert the contact angle (measured at the edge) to the height-to-radius ratio of the nanopocket (i.e., aspect ratio that is measured according to the profile). By this, (S5.7) can be rewritten as

$\left( \frac{h}{a} \right)^{4}+4\left( \tilde{\gamma}_{\mathrm{gl}}+\mathcal{T}_{\mathrm{pre}} \right)\left( \frac{h}{a} \right)^{2}-\Delta\tilde{\gamma}\cong0$, (S5.8)

where $\tilde{\gamma}_{\mathrm{gl}}=\frac{\gamma_{\mathrm{gl}}}{E_{2D}}$, $\Delta\tilde{\gamma}=\frac{2\gamma_{\mathrm{gl}}-\gamma_{\mathrm{gg}}}{E_{2D}}$, $\mathcal{T}_{\mathrm{pre}}=\frac{T_{\mathrm{pre}}}{E_{2D}}$. From the simple form of (S5.8) we can find a number of important implications.

- Solutions exist when $\Delta\tilde{\gamma}>0$.

Physically, $\Delta\tilde{\gamma}$ is the change of interfacial energies for the formation of a nanopocket; It involves the gain of two graphene-liquid interfaces and the loss of the graphene-graphene interface. The formation of nanopockets is energetically favorable only when it reduces the total (interfacial) energies, i.e., $\Delta\tilde{\gamma}>0$. We can then solve (S5.8) and find out the equilibrated aspect ratio:

$\left( \frac{h}{a} \right)^{2}=-2\mathcal{T}_{\mathrm{eff}}+\sqrt{4\mathcal{T}_{\mathrm{eff}}^{2}+\Delta\tilde{\gamma}}$, (S5.9)

where $\mathcal{T}_{\mathrm{eff}}=\tilde{\gamma}_{\mathrm{gl}}+\mathcal{T}_{\mathrm{pre}}$, implying that the graphene-liquid interface tension works against the elastic deformation like a pretension.

- Surface energies dominate pretension.

When the pretension is trivial, the aspect ratio of nanopockets is controlled mainly by the competition between surface energies and the strain energies associated with the out-of-plane deformation. The term $\tilde{\gamma}_{\mathrm{gl}}$ has been elusive to experimental characterizations but we may reasonably assume $\mathcal{T}_{\mathrm{pre}}{\ll\tilde{\gamma}}_{\mathrm{gl}}\sim\Delta\tilde{\gamma}\ll1$. This assumption leads to

$\left( \frac{h}{a} \right)_{\mathcal{T}_{\mathrm{pre}}\ll\Delta\tilde{\gamma}}=\Delta\tilde{\gamma}^{1/4}$, (S5.10)

which is consistent with previous works that have been derived via different analytical methods^7, 8^. This scaling relation has been used to explain the constant aspect ratios in substrate-supported 2D material bubbles^7, 8^.

- Pretension dominates surface energies.

In this limit, we assume $\tilde{\gamma}_{\mathrm{gl}}\sim\Delta\tilde{\gamma}\ll\mathcal{T}_{\mathrm{pre}}\ll1$ and simplify (S5.9) to

$\left( \frac{h}{a} \right)_{\mathcal{T}_{\mathrm{pre}}\gg\Delta\tilde{\gamma}}=\left( \frac{\Delta\tilde{\gamma}}{\mathcal{T}_{\mathrm{pre}}} \right)^{1/2}$. (S5.11)

The aspect ratio of nanopockets is controlled by the interplay of surface energies and the strain energies associated with both the pretension and the out-of-plane deformation. Equation (S5.11) predicts that the pretension would decrease the aspect ratio of a pocket, which has been observed in a similar but larger-scale system – a micron-sized drop capped between a substrate and a thin polymer film^10^.

- Changing pretension.

In our experiments, the pretension is applied by pressurizing the tBLG drumhead of radius $A$ with a height of $H$. We focused on nanopockets located near the center of the drumhead where the pretension $\mathcal{T}_{\mathrm{pre}}$ scale as $H^{2}/A^{2}$.^7^ The system is more likely to be in an intermediate state or not too far from the surface-energy-dominated state. In this case, we have $\tilde{\gamma}_{\mathrm{gl}}\sim\Delta\tilde{\gamma}\sim\left( h/a \right)_{0}^{4}\ll1$ and $\mathcal{T}_{\mathrm{pre}}\ll1$, where we define $\left( h/a \right)_{0}$ by the aspect ratio of nanopockets when the pretension is absent, i.e., $\mathcal{T}_{\mathrm{pre}}\ll\Delta\tilde{\gamma}$ or $H\ll A$. We then neglect the $\tilde{\gamma}_{\mathrm{gl}}$ term in (S5.8) and have

$\left( \frac{h}{a} \right)^{4}+4\mathcal{T}_{\mathrm{pre}}\left( \frac{h}{a} \right)^{2}-\Delta\tilde{\gamma}\cong0$. (S5.12)

We expect that (S5.12) becomes invalid when the pretension is too small to suppress the elastic instabilities associated with the radially inward displacement. It is then important to point out the criterion for the formation of instabilities, which is often equivalent to the condition under which the hoop stress at the pocket edge becomes negative, namely

$\left( \frac{h}{a} \right)^{2}\geq\left( \frac{h}{a} \right)_{\mathrm{cr}}^{2}=4\mathcal{T}_{\mathrm{pre}}$, (S5.13)

where we used (S5.5) and (S5.6). To verify the geometric relation in (S5.12) and its applicable domain given by (S5.13), we perform a more rigorous numerical analysis.

**Supplementary Figure 19.** (a) The critical aspect ratio of nanopockets for the initiation of elastic instabilities. The solid line is predicted by the simple analysis in (S5.13), which slightly deviates from the numerical results (circular markers) obtained according to the technique outlined in Section 4. The dashed line, therefore, is proposed (based on the fitting of numerical results) to describe the $\left( h/a \right)_{\mathrm{cr}}^{2}-\mathcal{T}_{\mathrm{pre}}$ relation. (b) The dependency of the aspect ratio of the nanopockets on the pretension for various $\Delta\tilde{\gamma}$. Solid markers are numerical results based on theories without considering the instability. Solid lines come from (S5.11). These results are valid in the blank region as aspect ratios in the shaded region meet the criterion for the instability formation.

### **6. Detailed Calculations**

**Supplementary Figure 20.** The schematic of a nanopocket with the inner and outer position of the wrinkled zone labeled.

**i) Before instabilities initiate**

As the out-of-plane deflection of these nanopockets is much larger than the (nominal) thickness of graphene, the analysis is based on FvK equations without a negligible bending rigidity, a.k.a. the membrane limit of FvK equations^10, 11, 12^. We begin by discussing the instability-free state.

The membrane limit dictates the out-of-plane equilibrium by

$\phi w^{'}=-\frac{1}{2}pr^{2}$, (S6.1)

and the compatibility by

$r\phi^{''}+\phi^{'}-\frac{\phi}{r}=-\frac{1}{2}{{E_{2D}w}^{'}}^{2}$, (S6.2)

where $w'$ denotes the differentiation with respect to $r$; $\phi$ is Airy stress function, based on which the stress resultants can be expressed,

$N_{r}=\frac{\phi}{r}$ and $N_{\theta}=\phi^{'}$*.* (S6.3)

Three boundary conditions are required to solve (S6.1) and (S6.2). Specifically, the in-plane displacement vanishes at the center, and the out-of-plane displacement vanishes at the edge:

$\lim_{r\to0} u\left( r \right)=0$ and $w\left( a \right)=0$. (S6.4)

Note that the first condition regarding $u$ can be related to Airy stress function by the hoop strain and Hooke’s law. The continuity of displacement and radial stress across the nanopocket edge offer two additional conditions, which are equivalent to the continuity of both stresses:

$\frac{\phi(a)}{a}=\frac{A}{a^{2}}+T_{\mathrm{pre}}$ and $\phi^{'}\left( a \right)=-\frac{A}{a^{2}}+T_{\mathrm{pre}}$. (S6.5)

The extra condition in (S6.4) and (S6.5) could be used to determine the constant $A$. Lastly, the pressure in the nanopocket is not known a priori; We determine it via the force balance at the contact line^[[2]](#footnote-2)^:

$\cos\beta_{0}=1-\frac{\Delta\gamma}{N_{r}(a)}$. (S6.6)

We introduce the following non-dimensionlization,

$\rho=\frac{r}{a}, W=\frac{w}{a}, H=\frac{h}{a}\mathcal{, u=}\frac{u}{a}, P=\frac{pa}{E_{2D}}, \Phi=\frac{\phi}{E_{2D}a}, \tilde{N}_{\rho}=\frac{N_{r}}{E_{2D}},\tilde{N}_{\theta}=\frac{N_{\theta}}{E_{2D}}$. (S6.7)

The dimensionless form of the problem (S6.1&S6.2) becomes

$\Phi W^{'}=-\frac{1}{2}P\rho^{2}$ (S6.8)

and

$\rho\Phi^{''}+\Phi^{'}-\frac{\Phi}{\rho}=-\frac{1}{2}{W^{'}}^{2}$. (S6.9)

They are to be solved subject to the boundary conditions

$\lim_{\rho\to0} \left[ \rho\Phi^{'}\left( \rho\right)-\nu\Phi\left( \rho\right) \right]=0, W\left( 1 \right)=0, \Phi\left( 1 \right)+\Phi^{'}\left( 1 \right)=2\mathcal{T}_{\mathrm{pre}}, \frac{1}{\left[ 1+{W^{'}}^{2}\left( 1 \right) \right]^{1/2}}=1-\frac{\Delta\tilde{\gamma}}{\Phi\left( 1 \right)},$ (S6.10)

where the first and second conditions come from (S6.4), the third combines (S6.5), and the last originates from (S6.6). Apparently, $\mathcal{T}_{\mathrm{pre}}$ and $\Delta\tilde{\gamma}$ control the system.

In Fig. S19, we showed the calculated results based on (S6.8-S6.10). Our prime interest is the dependency of the aspect ratio of nanopockets on $\mathcal{T}_{\mathrm{pre}}$ and $\Delta\tilde{\gamma}$ as well as the criterion for the formation of elastic instabilities, which have been predicted analytically in (S6.12) and (S6.14), respectively. Though these simple analytical predictions fail to fit the numerical results exactly, they provide good qualitative trends; Besides, we find excellent quantitative agreements after slight modifications to these analytical expressions, namely

$\left( S6.12 \right)\to\left( \frac{h}{a} \right)^{4}+2\mathcal{T}_{\mathrm{pre}}\left( \frac{h}{a} \right)^{2}-\Delta\tilde{\gamma}\cong0$ and $\left( S6.14 \right)\to\left( \frac{h}{a} \right)^{2}\geq\left( \frac{h}{a} \right)_{\mathrm{cr}}^{2}\cong3.6\mathcal{T}_{\mathrm{pre}}$. (S6.11)

The modified expressions in (S6.11) are adopted in the main text. We note that $\mathcal{T}_{\mathrm{pre}}\cong H^{2}/2A^{2}$ around the center of the host graphene drumhead of radius $A$ and height $H$.^[[3]](#footnote-3)^ In the main text, we rewrite (S6.11) in terms of geometric parameters in order to compare to experimental measurements, namely

$\left( \frac{h}{a} \right)^{4}+\left( \frac{H}{A} \right)^{2}\left( \frac{h}{a} \right)^{2}\cong\left( \frac{h}{a} \right)_{0}^{4}$ (S6.12)

and

$\left( \frac{h}{a} \right)^{2}\geq\left( \frac{h}{a} \right)_{\mathrm{cr}}^{2}\cong1.8\left( \frac{H}{A} \right)^{2}$, (S6.13)

where $\left( h/a \right)_{0}$ is defined by the aspect ratio of nanopockets when the pretension is negligible (i.e., $H\ll A$).

**ii) After instabilities initiate**

We consider the aspect ratio of the nanopockets when elastic instabilities start to propagate inward and outward. The origin of instabilities is the hoop compression as the ultra-lubricated nature of the twisted graphene-graphene interface allows the graphene in $r\geq a$ to slide inward radially in response to the pressure in $0\leq r\leq a$. Instabilities appear when the pretension is relatively small. The thinness of graphene makes the tension field theory particularly appropriate to describe the stress state of the sheet within the wrinkled region (the shaded region in Fig. S19). Similar to the poking problem in Ref.^11, 12, 13^; the bulging problem includes a number of regions: pressurized and unwrinkled region $[0,R_{I}]$, pressurized and wrinkled region $[R_{I},a]$, unpressurized and wrinkled region $[a,R_{O}]$, and the outmost unpressurized and unwrinkled region $[R_{O},\infty]$. We present the governing equations, together with the boundary and matching conditions, in dimensionless forms for these domains inside and out.

- $\rho\in[0,\rho_{I}]$

Equations (S6.8) and (S6.9) still hold. The inner boundary condition comes from the in-plane displacement at the center, or equivalently

$\lim_{\rho\to0} \left[ \rho\Phi^{'}\left( \rho\right)-\nu\Phi\left( \rho\right) \right]=0$. (S6.14)

- $\rho\in[\rho_{I},1]$

We use tension field theory (TFT), assuming that thin objects cannot resist any compressive forces. This assumption allows the exact solution to the FvK equations, namely

$\tilde{N}_{\theta}=0, \tilde{N}_{\rho}=\frac{C_{I}}{\rho}, W=\frac{P}{6C_{I}}(1-\rho^{3})$, (S6.15)

where we enforced $W\left( 1 \right)=0$ and the continuity of $W^{'}$ at $\rho=\rho_{I}$. We introduced two unknowns ($C_{I}$ and $\rho_{I}$) as well as brought three continuity conditions:

$\Phi^{'}\left( \rho_{I} \right)=0, \Phi\left( \rho_{I} \right)=C_{I}, W(\rho_{I})=\frac{P}{6C_{I}}(1-{\rho_{I}}^{3})$. (S6.16)

- $\rho\in[1,\rho_{O}]$

We use the planar TFT, which shares the same solution to stresses as (S6.15),

$\tilde{N}_{\theta}=0$ and $\tilde{N}_{\rho}=\frac{C_{I}}{\rho}$, (S6.17)

where the condition of $\tilde{N}_{\rho}\left( 1^{-} \right)=\tilde{N}_{\rho}\left( 1^{+} \right)$ has been satisfied.

- $\rho\in[\rho_{O},\infty]$

In this region, the regular Lame solution can be used,

$\tilde{N}_{\rho}=\frac{B}{\rho^{2}}+\mathcal{T}_{\mathrm{pre}}$ and $\tilde{N}_{\theta}=-\frac{B}{\rho^{2}}+\mathcal{T}_{\mathrm{pre}}$*.* (S6.18)

The constant $B$ can be determined by the fact of $\tilde{N}_{\theta}\left( \rho_{O} \right)=0$, namely

$B=\rho_{O}^{2}T_{\mathrm{pre}}$. (S6.19)

The continuity of radial stress across the outer wrinkling tip can give an equation for $\rho_{O}$,

$C_{I}=2\rho_{O}\mathcal{T}_{\mathrm{pre}}$. (S6.20)

The problem now contains 8 unknowns (3 for two coupled ODEs in the innermost region, the pressure $P$, the constants $C_{I}$ and $B$, and the lengths $\rho_{I}$ and $\rho_{O}$). 6 of the required conditions to solve the problem have been given by (S6.11), (S6.16), (S6.19), and (S6.20). The force balance at the contact line in (S6.6) should hold. The final condition comes from the in-plane displacement across the wrinkled regions; We use the kinematics and Hooke’s law to show that

$u\left( \rho_{O} \right)-u\left( \rho_{I} \right)=\int_{\rho_{I}}^{1} \epsilon_{\rho}d\rho+\int_{1}^{\rho_{O}} \epsilon_{\rho}d\rho=\int_{\rho_{I}}^{1} \left[ \frac{C_{I}}{\rho}-\frac{1}{2}\left( \frac{1}{2}\frac{P\rho^{2}}{C_{I}} \right)^{2} \right]d\rho+\int_{1}^{\rho_{O}} \frac{C_{I}}{\rho}d\rho$, (S6.21)

which further leads to

$-2\nu\rho_{O}\mathcal{T}_{\mathrm{pre}}+\nu C_{I}-C_{I}\log\frac{\rho_{O}}{\rho_{I}}+\frac{1}{40}\frac{P^{2}}{C_{I}^{2}}\left( 1-\rho_{I}^{5} \right)=0$. (S6.22)

We use Matlab ODE solver (bvp5c) to calculate (S6.14-S6.22) and show the results in Fig. S21. The predicated aspect ratios of nanopockets start to deviate from (S6.11) (that does not consider the instability) after the criterion in (S6.11) is satisfied. In particular, in the shaded area in Fig. S21, we find that the release of the (compressive) stress by instability causes increased aspect ratios. The underestimation of (S6.11) is around 20% when $\mathcal{T}_{\mathrm{pre}}\sim{10}^{-5}$.

**Supplementary Figure 21.** The dependency of the aspect ratio of the nanopockets on the pretension ($\Delta\tilde{\gamma}=0.0001$ in our calculations). The red line is due to (S6.11), while the black line is calculated based on theories considering instabilities. The dashed line draws the criterion for the formation of instabilities, which is from (S6.11).

### **References**

1. He, K. T., Wood, J. D., Doidge, G. P., Pop, E., Lyding, J. W. Scanning tunneling microscopy study and nanomanipulation of graphene-coated water on mica. *Nano Lett.* **12**, 2665-2672 (2012).

2. Kuwabara, M., Clarke, D. R., Smith, D. A. Anomalous superperiodicity in scanning tunneling microscope images of graphite. *Appl. Phys. Lett.* **56**, 2396-2398 (1990).

3. Tan, Z., et al. Building large-domain twisted bilayer graphene with van hove singularity. *ACS Nano* **10**, 6725-6730 (2016).

4. Carozo, V., Almeida, C. M., Ferreira, E. H., Cancado, L. G., Achete, C. A., Jorio, A. Raman signature of graphene superlattices. *Nano Lett.* **11**, 4527-4534 (2011).

5. Tuinstra, F. Raman Spectrum of Graphite. *J. Chem. Phys.* **53**, 1126-1130 (1970).

6. Maultzsch, J., Reich, S., Thomsen, C. Double-resonant Raman scattering in graphite: Interference effects, selection rules, and phonon dispersion. *Phys. Rev. B* **70**, 2806-2810 (2004).

7. Dai, Z., et al. Interface-governed deformation of nanobubbles and nanotents formed by two-dimensional materials. *Phys. Rev. Lett.* **121**, 266101 (2018).

8. Sanchez, D. A., et al. Mechanics of spontaneously formed nanoblisters trapped by transferred 2D crystals. *Proc. Natl. Acad. Sci. U. S. A.* **115**, 7884-7889 (2018).

9. Timoshenko, S. P., Goodierwrited, J. N. *Theory of Elasticity.3rd Ed*. McGraw-Hill Book Company (1970).

10. Schulman, R. D., Dalnoki-Veress, K. Droplets capped with an elastic film can be round, elliptical, or nearly square. *Phys. Rev. Lett.* **121**, 248004 (2018).

11. Vella, D., Davidovitch, B. Regimes of wrinkling in an indented floating elastic sheet. *Phys. Rev. E* **98**, 013003 (2018).

12. Dai, Z., Sanchez, D. A., Brennan, C. J., Lu, N. Radial buckle delamination around 2D material tents. *J. Mech. Phys. Solids* **137**, 103843 (2020).

13. Davidovitch, B., Vella, D. Partial wetting of thin solid sheets under tension. *Soft Matter* **14**, 4913-4934 (2018).

1. This appears like an elastic version of Young’s equation developed for wetting problems, which could be derived according to the boundary terms arising from the principle of minimum free energy. [↑](#footnote-ref-1)
2. We neglect the effect of the graphene-liquid interface tension to be consistent with the out-of-plane equilibrium in (S6.1). This treatment would be appropriate only for nanopockets with small aspect ratios, say $h/a\ll1$; In Section 5 we suggest that all surface energies scale as $E_{2D}\left( h/a \right)^{4}$; The contribution of the graphene-liquid surface tension to the pressure scales as $\gamma_{\mathrm{gl}}\kappa\sim E_{2D}h^{5}/a^{6}$, which is negligible compared with the contribution of the sheet tension scales as $N_{r}\kappa\sim E_{2D}h^{3}/a^{4}$. [↑](#footnote-ref-2)
3. This approximation works well for the top layer of the tBLG (even after wrinkling occur in the suspended region) as its edge is supported by the bottom layer. We expect a transverse asymmetry due to the different stress levels in the two layers, which may slightly modify our simple expressions that are based on the symmetry argument. [↑](#footnote-ref-3)
