# Supplementary material for: Elastocapillary cleaning of twisted bilayer graphene interfaces
Source: Nat Commun. 2021 Aug 20;12:5069. doi: 10.1038/s41467-021-25302-2 (PMC8379234; doi:10.1038/s41467-021-25302-2)
Supplement: Supplementary file 3 — Description of Additional Supplementary Files [file 41467_2021_25302_MOESM3_ESM.docx]

Description of Additional Supplementary Files

Title: Supplementary Movie 1.

Description: Spontaneous coalescence of nanopockets.
